# Potential Elevation Shift of the European Beech Stands (*Fagus sylvatica* L.) in Serbia

**DOI:** 10.3389/fpls.2019.00849

**Published:** 2019-07-09

**Authors:** Lazar Pavlović, Dejan Stojanović, Emina Mladenović, Milena Lakićević, Saša Orlović

**Affiliations:** ^1^Faculty of Agriculture, University of Novi Sad, Novi Sad, Serbia; ^2^Institute of Lowland Forestry and Environment, University of Novi Sad, Novi Sad, Serbia

**Keywords:** European beech, potential distribution, climate change, biomod2, SDMs

## Abstract

According to climate projection models, the global temperature is expected to rise by at least 1.5°C by the end of this century. According to some studies the expected rise in Serbia is even higher. Global warming may result in creating new areas for forest growth. Although creating new forests would be a positive outcome in some areas, global warming can cause negative impacts in other areas, and this can lead to forest loss and the shift of geographical ranges, or even extinction, of plant species. The European beech is the dominant forest tree species in Serbia, featuring high ecological importance and economic value. In mixed or pure stands, beech forests cover approximately 660,400 ha, accounting for 29.3% of the total Serbian forest area. In the present study, the effects of climate change on the distribution of the European beech stands in Serbia, with an emphasis on their elevation shifts, were examined using species distribution models (SDMs). Data for the present tree cover in Serbia, climate projections, and environmental data were used for model building. The models were first tested against present inventory data. In these tests, the models were found to provide accurate projections, as shown by their true skills statistics (TSS) values ranging from 0.652 to 0.736 and area under the curve (AUC) values ranging from 0.868 to 0.937. The potential distribution patterns predicted by the models indicate that the European beech elevational distribution in Serbia would decrease, exhibiting a significant upward shift in elevation during the first part of this century. Current beech stand locations could be changed, and other areas at higher elevations may be more suitable for beech growth. After 2071, European beech stands at elevations below 500 m would be even smaller. This change is caused by temperature rise and occurrence of climate extremes. However, on the highest elevations, further upward shift of the species is not expected.

## Introduction

Over the last 50 years, climate change has been affecting forest ecosystems globally, and climate projection models argue that its impact will increase by the end of this century (Vose et al., 2012; Grimm et al., 2013; Brandt et al., 2016). The expected rise of the global temperature will create new forest areas, particularly in northern zones and at higher elevations. Moreover, increased carbon dioxide concentrations in the atmosphere will further accelerate forest growth (Silva et al., 2016). Although creating new forests is a beneficial outcome of climate change in some areas, other areas would, in contrast, be exposed to high temperature extremes, drought, wildfire, etc., leading to forest loss and the shift of geographical areas or even extinction of plant species (IPCC and Core Writing Team, 2014; Tian et al., 2016). In order to preserve forest ecosystems, many countries are developing strategies and policies to reduce the risks caused by climate change and to seize the opportunities arising from climate change adaptations (Bosworth et al., 2008; Joyce et al., 2009; Littell et al., 2011; Janowiak et al., 2014). Such adaptations are intrinsically related to the management of forest ecosystems, encompassing the determination of climate change impact on an area; to the estimated species’ or ecosystem’s sensitivity to the projected impacts; as well as to adaptation strategies and their incorporation in the forest ecosystem (Cross et al., 2012, 2013; Stein et al., 2014; Brandt et al., 2016).

Species distribution models (SDMs) are useful tools for developing strategies and adaptation policies to climate change. SDMs create a statistical relationship between the current climate and the occurrence of the species. They use a number of statistical approaches to find relationships and gridded environmental data to extrapolate model projections in space and time (Elith and Leathwick, 2009; Franklin and Miller, 2010; Thuiller and Münkemüller, 2010; Serra-Diaz et al., 2012; Hijmans and Elith, 2017).

The European beech (*Fagus sylvatica* L.) is the dominant deciduous tree species in Central Europe and in the higher elevated areas of Southern Europe (Bolte et al., 2016; Mausolf et al., 2018). Owing to its high ecological importance and economic value, the European beech has a significant role in the European forestry sector and the overall European forest biodiversity. According to Banković et al. (2009), beech forests, in both mixed and pure stands, cover approximately 660,400 ha in Serbia, accounting for 29.3% of the total Serbian forest area. As the dominant deciduous tree species, it is widely distributed in Serbia. It can be found under different climate conditions (mostly mild winters and moist summers) and at a wide variety of sites with the exception of extremely dry soils featuring a low water storage capacity, stagnic soils, or soils prone to flooding and high groundwater table (Ellenberg and Leuschner, 2010; Bolte et al., 2016). In the future, the increasing variability of climate and the frequency of climatic extremes (IPCC and Core Writing Team, 2014) will impact tree growth (Easterling et al., 2000; Anderegg et al., 2015). A number of studies (Köcher et al., 2009; Zang et al., 2014; Zimmermann et al., 2015; Kunz et al., 2018) consider the European beech as a species sensitive to climatic extremes, especially drought and water deficit, which reduces its competitive advantage over less drought-sensitive species, and this will ultimately result in landscape transformation (Scharnweber et al., 2011; Barigah et al., 2013; Urli et al., 2013). Consequently, the geographical range of the European beech is expected to decrease in the future (Thurm et al., 2018), accompanied by an upward elevation shift of the species. At the present, higher elevations (exceeding 1,200 m) are not favorable to beech growth due to lower temperatures and late frosts (Budeanu et al., 2016; Kolář et al., 2017). According to the national reports on the climate and climate change in the Republic of Serbia (DRINKADRIA project, 2014), the average temperature in Serbia is expected to rise by approximately 3.7°C by the end of the century (the A2 climate change scenario), so it is expected that beech growth will be viable at higher elevations. However, certain species, such as Norway spruce, will be adversely affected by the forecasted climate change and will become sparser at such elevations (Falk and Hempelmann, 2013).

In the present study, we hypothesize that the European beech area shifts upward in elevation under changing climate conditions. SDMs were used to test the hypothesis in Southeastern Europe in Serbia.

## Materials and Methods

### Study Area

The area of this study was the territory of Republic of Serbia situated in Southeastern Europe, in the center of the Balkan Peninsula and the southern part of the Pannonian Plain (between 41°53′ and 46°11′ latitude north and 18°49′ and 23°00′ longitude east). Available data in the National Forest Inventory used in the study covers 77,474 km^2^, while forests cover amount 22370.17 km^2^, i.e., 25.3% of the territory of Serbia.^1^

### Presence/Absence Data

**FIGURE 1:**
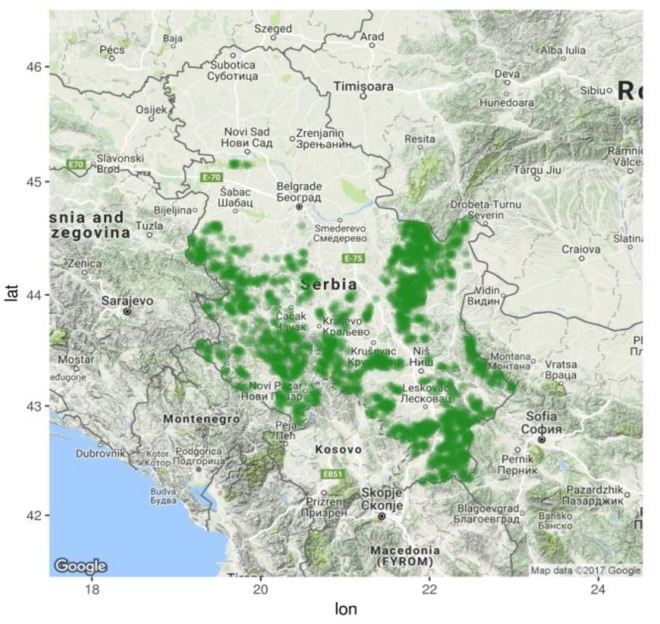
Distribution of European beech forests in Serbia according to the National Forest Inventory data (Banković et al., 2009) obtained in R packages “RgoogleMaps” (Loecher and Ropkins, 2015) and “mapproj” (McIlroy, 2018).

Records of the presence/absence of the European beech (*F. sylvatica* L.) in Serbia were obtained from the National Forest Inventory of Serbia (Figure 1; Banković et al., 2009). These records contain the data for 19,371 tested cells distributed in the 2 × 2-km network (grid), out of which 5,852 were identified as forest cells. Beech was found in 1,651 forest cells, i.e., in an area covering more than 28% of the Serbian forest area. Therefore, beech can be considered as the dominant forest species in Serbia. Beech forests are found in the mountainous regions of Serbia in both pure stands and mixed stands with broadleaves and conifers. Their distribution ranges from 100 to 1,700 m.

The data set was split randomly into 80% training and 20% evaluating subsets. The splitting was stratified in order to keep the data on the presence/absence of the European beech in Serbia equal in the training and the evaluating data subsets. The splitting was carried out using the caret R package (Kuhn, 2008).

### Environmental Data Sets

A set of 29 bioclimatic variables was used for both the current and the projected future climate (Table 1). Bioclim variables were downloaded from the CliMond Archive (Kriticos et al., 2012). The bioclimatic variables were based on the monthly temperature and rainfall values. Those values were used in order to generate biologically meaningful variables, which are relevant for the distribution of beech as they represent annual trends (e.g., the mean annual temperature), seasonality (e.g., the annual range in temperature and precipitation), and extreme or limiting environmental factors. A resolution of 10 arcmin, which corresponds to 18.6 × 18.6 km, was employed for the variables, as well as the MIROC-H A2 climate projections.

**Table 1:** Bioclimatic and soil variables used in the study (Hutchinson et al., 2009; Kriticos et al., 2014; Hengl et al., 2017).

Variable number	Variable
Bio01	Annual mean temperature (°C)
Bio02	Mean diurnal temperature range [mean(period max–min)] (°C)
Bio03	Isothermality (Bio02/Bio07)
Bio04	Weekly temperature seasonality (C of V)
Bio05	Maximum temperature of the warmest week (°C)
Bio06	Minimum temperature of the coldest week (°C)
Bio07	Annual temperature range (Bio05–Bio06) (°C)
Bio08	Mean temperature of the wettest quarter (°C)
Bio09	Mean temperature of the driest quarter (°C)
Bio10	Mean temperature of the warmest quarter (°C)
Bio11	Mean temperature of the coldest quarter (°C)
Bio12	Total annual precipitation (mm)
Bio13	Precipitation of the wettest week (mm)
Bio14	Precipitation of the driest week (mm)
Bio15	Weekly precipitation seasonality (C of V)
Bio16	Precipitation of the wettest quarter (mm)
Bio17	Precipitation of the driest quarter (mm)
Bio18	Precipitation of the warmest quarter (mm)
Bio19	Precipitation of the coldest quarter (mm)
Bio20	Annual mean radiation (W m^-2^)
Bio21	Highest weekly radiation (W m^-2^)
Bio22	Lowest weekly radiation (W m^-2^)
Bio23	Annual mean moisture index
Bio24	Highest weekly moisture index
Bio25	Lowest weekly moisture index
Bio26	Weekly moisture index seasonality (C of V)
Bio27	Mean moisture index of the wettest quarter
Bio28	Mean moisture index of the driest quarter
Bio29	Mean moisture index of the warmest quarter
Soil 1	Depth to bedrock (R horizon) up to 200 (cm)
Soil 2	Absolute depth to bedrock (cm)
Soil 3–Soil 6	Bulk density (fine earth) (kg/m^3^)
Soil 7–Soil 10	Clay content [0–2 μm mass fraction (%)]
Soil 11–Soil 14	Coarse fragments volumetric fraction (%)
Soil 15–Soil 18	Silt content [2–50 μm mass fraction (%)]
Soil 19–Soil 22	Sand content [50–2,000 μm mass fraction in (%)]
Soil 23–Soil 26	Cation exchange capacity of soil (cmol_c_/kg)
Soil 27–Soil 30	Soil organic carbon content (fine earth fraction) (g/kg)
Soil 31–Soil 34	Soil pH × 10 in H_2_O
Soil 35–Soil 38	Soil pH × 110 in KCl


*The soil variables 3–38 describe the soil conditions at the following four respective soil layers: 0–15, 15–30, 30–60, and 60–100 cm.*

In order to develop high-precision distribution models, it is important to include soil characteristics in the modeling (Seynave et al., 2008). In addition to the climatic variables, a set of 38 soil variables were used in the study (Table 1). The first two soil variables describe the soil depth, and for the other soil characteristics, four separate variables were used for the corresponding four soil depths (Table 1). All of the soil variables used were obtained from the soilgrids.org database using a resolution of 250 m (Hengl et al., 2017).

Elevation maps were derived from the Copernicus Land Monitoring System at a resolution of 25 m. The catdes function of the FactorMineR package (Lê et al., 2008) was used for characterizing variable importance for determining the presence/absence of beech. FactoMineR is R package for multivariate exploratory data analysis. It performs classical principal component analysis (PCA), correspondence analysis (CA), multiple correspondence analysis (MCA), clustering, as well as advanced analyses including different data structures. The model to be used in assessing the climatic change impacts and soil characteristics on the presence of the beech was designed with variables in which *p* was less than 0.01 (*p* < 0.01) (Husson et al., 2010). Both single and ensemble SDMs were employed in the study. The ensemble platform biomod2 (Thuiller et al., 2009) offers the possibility of running 10 modeling techniques for the species distribution modeling used to predict the potential distribution of the European beech. To enhance computation efficiency and minimize computation time requirements, the following seven algorithms were applied: generalized linear models (GLMs), generalized boosting model (GBM), classification tree analysis (CTA), artificial neural networks (ANNs), flexible discriminant analysis (FDA), multivariate adaptive regression spline (MARS), and random forest for classification and regression (RF). Although certain models such as the generalized additive model (GAM), MAXENT.Phillips, and Maximum Entropy MAXENT.Tsuruoka produce good results, they were disregarded in our study for being overly time-consuming in our case and featuring high computer performance requirements.

Owing to substantial discrepancies between the results obtained with different models, the ensemble model was considered as the most suitable for minimizing the limitations of single models and producing high-precision results (Coetzee et al., 2009). The performance of each model was assessed using receiver operating characteristic (ROC) known as area under the curve (AUC) and true skills statistic (TSS) values. TSS values were employed because in it, at the range from -1 to 1, either omission or commission errors are not affected by prevalence of the finding under consideration, as opposed to Kappa values (Allouche et al., 2006). According to Gama et al. (2016), AUC values can be random (<0.5), poor (0.5–0.7), good (fair) (0.7–0.9), and excellent (>0.9). Gama et al. (2016) also labels TSS values as poor (0.2–0.5), useful (0.6–0.8), and good to excellent (>0.8).

## Results

### Test of the Models

For the present climatic conditions, the models showed a good fit between the observed and the predicted presence/absence (Table 2). The TSS values obtained ranged from 0.652 to 0.736, which is considered as good. A better model performance was indicated by the AUC values ranging from 0.868 to 0.937, which is considered as excellent. Among the single models, RF, GLM, and GBM exhibited the best performance (Table 2).

**Table 2:** Evaluation of European beech (*F. sylvatica* L.) distribution models by TSS and by the area under the ROC curve (AUC).

Model	TSS	AUC
GLM	0.692	0.923
RF	0.736	0.937
FDA	0.703	0.913
CTA	0.652	0.868
ANN	0.682	0.911
MARS	0.693	0.919
GBM	0.700	0.922
ENS	0.734	0.923


*The models were tested with their projections for the presence/absence of European beech in Serbia. For the acronyms of the models, see section “Materials and Methods.”*

However, the fundamental feature of the biomod2 package is the ability to combine the projection of single models and build ensemble models based on the single models. The results obtained show that the AUC of the present ensemble model (ENS) was 0.923, which is a bit lower than that obtained using the RF model, but still better than the results obtained using the other models (Table 2).

### Projections for the Periods 2041–2070 and 2071–2100 Under the Climatic Scenario A2

**FIGURE 2:**
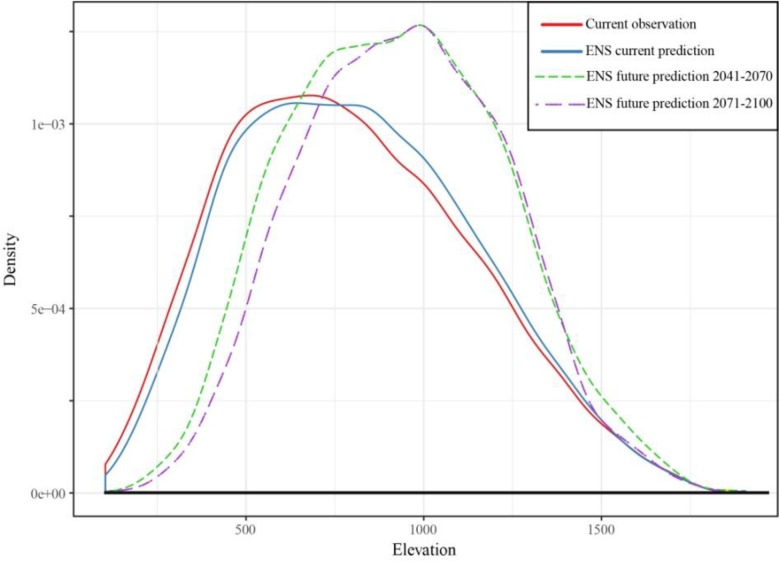
Projections of SDMs for the distribution of European beech along the elevation gradient for the current climate (solid blue curve) and the climate predicted by the A2 scenario from 2041 to 2070 and from 2071 to 2100 (dashed curves). The solid red curve indicates the present observed distribution. Ensemble models (ENS) were used for the projections of beech distribution. Figure obtained in R package “ggplot2” (Wickham, 2016).

The projected distribution of the European beech stands in Serbia, obtained using the ensemble model and the biomod2 package, indicated that the current beech forest cover will change in the future. Elevational distribution of the beech will be narrowed, and it will be shifting from lower to higher elevations. During 2041–2070, in lower elevations, an upward elevation shift is expected, accompanied by the increased density of the European beech stands at higher elevations (Figure 2). It can be noticed that the maximum density of the beech will be significantly higher in the future. Elevations between 800 and 1,100 m will be more suitable for beech growth than the elevations at which the European beech stands currently predominate in Serbia, i.e., 500–900 m (Figure 2).

After 2071, the projections obtained show that the European beech stands in Serbia found at lower elevations will become even sparser, especially at elevations lower than 500 m (Figure 2). The occurrence of beech stands at higher elevations is predicted to be similar as in the previous period (2041–2070), and the species will not shift further upward in elevation. At the highest elevations exceeding 1,400 m, it seems unlikely that beech will migrate above the current upper distribution limit (Figure 2).

## Discussion

The results of the present study show that the biomod2 platform is able to predict the current distribution of beech in Serbia. This suggests that it can be used also for predicting any future distribution changes caused by climatic change. The biomod2 platform has been used for such projections in a number of studies (Duque-Lazo et al., 2016; Takolander et al., 2019). In our study, this platform exhibited higher accuracy with regard to single models when tested against the beech distribution in the current climate. The AUC accuracy ranged from 0.868 to 0.937, considered as excellent, whereas the TSS accuracy ranged from 0.652 to 0.736, considered as good. Despite the high accuracy of the single models, the ensemble model forecasting is an effective method for aggregating single SDMs and resolving the problem of intermodel variability, thus leading to results of higher precision (Xu et al., 2015; Lei et al., 2017).

The model projections of the present study indicate that European beech areal in Serbia will probably change in the future, mostly in elevation, which will result in landscape transformation of mountain regions. Not only will temperature rise in the future in Serbia, but also the average precipitation is expected to decrease by 15%. The number of tropical and dry days (Kržič et al., 2011) will also increase, which may lead to the shift or extinction of species (Tian et al., 2016). Owing to climate change, many tree species are predicted to shift their geographic ranges (Takolander et al., 2019). As beech is very sensitive to drought, temperature extremes, and global warming, a number of studies (Scharnweber et al., 2011; Kunz et al., 2018) have suggested that extreme climate events, especially drought, can lead to beech forest loss. The results obtained in this study predict an upward beech elevation shift in lower altitudes. The beech shift to higher elevations and beech forest loss have been predicted in many studies (Dorado-Liñán et al., 2017; Sedmáková et al., 2019). Peñuelas et al. (2007) argued that the European beech will shift from lower to higher altitudes in some areas in Spain, accompanied by a decrease in its distribution range. Furthermore, it will replace heathlands and grasslands, as well as some coniferous forests, at higher elevations (Peñuelas et al., 2007). Kolář et al. (2017) concluded that even though average climate characteristics in the montane belt will be, in the future, more favorable for beech, its growth and vitality will be probably still limited by climate extremes, such as late frosts.

In spite of the shift in elevation, it seems unlikely that European beech in Serbia will migrate above current upper distribution limit at elevations exceeding 1,400 m during 2071–2100. This limitation of shifting can be attributed to the sensitivity of beech to drought and temperature rise (Kolář et al., 2017) as well as to shallow mountain soils with a low water storage capacity, which are usually present at higher elevations (Reif et al., 2017). The results obtained in the present study are consistent with the results of Seynave et al. (2008) and Calvaruso et al. (2017), who reported that beech growth is reduced by unfavorable soil properties, especially in shallow soils. However, an upward shift in elevation of the European beech is to be expected. The reason for such an elevation shift is probably the annual temperature rise. Mátyás et al. (2010) reported that a temperature rise of 1°C can lead to an upward species shift of approximately 170 m along the mountain slope. By comparing pictures taken at three time instants (1 year in the 1920s, 1943, and 2003), Peñuelas et al. (2007) demonstrated that both shifting of beech stands and changes in their density took place simultaneously with warming during the period examined. Yao and Zhang (2015) compared two mountain localities on the Tibetan Plateau with a difference in temperature and showed that the treeline occurred 500–1,000 m higher in the localities with a higher temperature. Moreover, with a temperature rise at higher elevations, soil decomposition will be facilitated (Gutiérrez-Girón et al., 2015) and more nutrients will be available for plant uptake. All these phenomena, accompanied by the phenotypic plasticity of the European beech, can lead to the shift of the species from lower to higher elevations.

## Conclusion

Using the biomod2 platform, we evaluated the performance of SDMs. As main highlights we found the following:

- Models showed good accuracy, and results of the TSS and AUC of the models ranged from good to excellent.
- In the middle (2041–2071) and late 21st century (2071–2100), it is expected that habitats of the European beech, which are at elevations between 900 and 1,100 m, will be more suitable for growth than the habitats between 500 and 900 m, at which the beech stands currently predominate in Serbia.
- A further upward shift of the beech at the highest elevations above 1,400 m is not expected in Serbia.

## Author Contributions

LP and DS conceived the presented idea. LP developed the theory and performed the computations. DS and SO verified the analytical methods. DS, ML, and EM encouraged LP to investigate and supervised the findings of this work. All authors discussed the results and contributed to the final manuscript.

## Conflict of Interest Statement

The authors declare that the research was conducted in the absence of any commercial or financial relationships that could be construed as a potential conflict of interest.

## Footnotes

1 https://www.cia.gov/library/publications/the-world-factbook/

## References

[B1] Allouche O. Tsoar A. Kadmon R. (2006). Assessing the accuracy of species distribution models: prevalence, kappa and the true skill statistic (TSS). *J. Appl. Ecol.* 43 1223–1232. 10.1111/j.1365-2664.2006.01214.x

[B2] Anderegg W. R. L. Schwalm C. Biondi F. Camarero J. J. Koch G. Litvak M. (2015). Pervasive drought legacies in forest ecosystems and their implications for carbon cycle models. *Science* 349 528–532. 10.1126/science.aab1833 26228147

[B3] Banković S. Medarević M. Pantić D. Petrović N. (2009). *National Forest Inventory of the Republic of Serbia.* Belgrade: Ministry of Agriculture, Forestry and Water management of the Republic of Serbia, Forest Directorate.

[B4] Barigah T. S. Charrier O. Douris M. Bonhomme M. Herbette S. Améglio T. (2013). Water stress-induced xylem hydraulic failure is a causal factor of tree mortality in beech and poplar. *Ann. Bot.* 112 1431–1437. 10.1093/aob/mct204 24081280 PMC3806533

[B5] Bolte A. Czajkowski T. Cocozza C. Tognetti R. de Miguel M. Pšidová E. (2016). Desiccation and mortality dynamics in seedlings of different European beech (*Fagus sylvatica* L). populations under extreme drought conditions. *Front. Plant Sci.* 7:751. 10.3389/fpls.2016.00751 27379105 PMC4906631

[B6] Bosworth D. Birdsey R. Joyce L. Millar C. (2008). Climate change and the nation’s forests: challenges and opportunities. *J. For.* 106 214–221.

[B7] Brandt L. A. Butler P. R. Handler S. D. Janowiak M. K. Shannon P. D. Swanston C. W. (2016). Integrating science and management to assess forest ecosystem vulnerability to climate change. *J. For.* 115 212–221. 10.5849/jof.15-147

[B8] Budeanu M. Petritan A. M. Flaviu P. Vasile D. Nicu Constantin T. (2016). The resistance of European beech (*Fagus sylvatica*) from the eastern natural limit of species to climate change. *Not. Bot. Horti. Agrobot. Cluj. Napoca.* 44 625–633. 10.15835/nbha44210262

[B9] Calvaruso C. Kirchen G. Saint-André L. Redon P.-O. Turpault M.-P. (2017). Relationship between soil nutritive resources and the growth and mineral nutrition of a beech (*Fagus sylvatica*) stand along a soil sequence. *Catena* 155 156–169. 10.1016/j.catena.2017.03.013

[B10] Coetzee B. W. T. Robertson M. P. Erasmus B. F. N. Van Rensburg B. J. Thuiller W. (2009). Ensemble models predict important bird areas in southern Africa will become less effective for conserving endemic birds under climate change. *Glob. Ecol. Biogeogr.* 18 701–710. 10.1111/j.1466-8238.2009.00485.x

[B11] Cross M. S. McCarthy P. D. Garfin G. Gori D. Enquist C. A. F. (2013). Accelerating adaptation of natural resource management to address climate change. *Conserv. Biol.* 27 4–13. 10.1111/j.1523-1739.2012.01954.x 23110636 PMC3562478

[B12] Cross M. S. Zavaleta E. S. Bachelet D. Brooks M. L. Enquist C. A. F. Fleishman E. (2012). The adaptation for conservation targets (ACT) framework: a tool for incorporating climate change into natural resource management. *Environ. Manage.* 50 341–351. 10.1007/s00267-012-9893-7 22773068 PMC3410031

[B13] Dorado-Liñán I. Akhmetzyanov L. Menzel A. (2017). Climate threats on growth of rear-edge European beech peripheral populations in Spain. *Int. J. Biometeorol.* 61 2097–2110. 10.1007/s00484-017-1410-5 28733860

[B14] DRINKADRIA project (2014). *Climate and Climate Change Data on National Level Republic of Serbia.* Belgrade: Institute for Development of Water Resources Available at: http://drinkadria.fgg.uni-lj.si/externalapp/content/climate/FB10_CC_Serbia_national.pdf

[B15] Duque-Lazo J. Gils H. Groen T. A. Navarro-cerrillo R. M. (2016). Transferability of species distribution models: the case of *Phytophthora cinnamomi* in southwest Spain and southwest Australia. *Ecol. Model.* 320 62–70. 10.1016/j.ecolmodel.2015.09.019

[B16] Easterling D. R. Meehl G. A. Parmesan C. Changnon S. A. Karl T. R. Mearns L. O. (2000). Climate extremes: observations, modeling, and impacts. *Science* 289 2068–2074. 11000103 10.1126/science.289.5487.2068

[B17] Elith J. Leathwick J. R. (2009). Species distribution models: ecological explanation and prediction across space and time. *Annu. Rev. Ecol. Evol. Syst.* 40 677–697. 10.1146/annurev.ecolsys.110308.120159

[B18] Ellenberg H. Leuschner C. (2010). *Vegetation Mitteleuropas Mit Den Alpen, 6.* Stuttgart: Verlag Eugen Ulmer.

[B19] Falk W. Hempelmann N. (2013). Species favourability shift in Europe due to climate change: a case study for *Fagus sylvatica* L. and *Picea abies* (L). Karst. based on an ensemble of climate models. *J. Climatol.* 18:787250 10.1155/2013/787250

[B20] Franklin J. Miller J. A. (2010). *Mapping Species Distributions: Spatial Inference and Prediction.* Cambridge: Cambridge University Press.

[B21] Gama M. Crespo D. Dolbeth M. Anastácio P. (2016). Predicting global habitat suitability for *Corbicula fluminea* using species distribution models: the importance of different environmental datasets. *Ecol. Model.* 319 163–169. 10.1016/j.ecolmodel.2015.06.001

[B22] Grimm N. B. Chapin F. S. III Bierwagen B. Gonzalez P. Groffman P. M. Luo Y. (2013). The impacts of climate change on ecosystem structure and function. *Front. Ecol. Environ.* 11 474–482. 10.1890/120282

[B23] Gutiérrez-Girón A. Díaz-Pinés E. Rubio A. Gavilán R. G. (2015). Both altitude and vegetation affect temperature sensitivity of soil organic matter decomposition in Mediterranean high mountain soils. *Geoderma* 23 1–8. 10.1016/j.geoderma.2014.08.005

[B24] Hengl T. Mendes de Jesus J. Heuvelink G. B. M. Ruiperez Gonzalez M. Kilibarda M. Blagotic A. (2017). SoilGrids250m: global gridded soil information based on machine learning. *PLoS One* 12:e0169748. 10.1371/journal.pone.0169748 28207752 PMC5313206

[B25] Hijmans R. J. Elith J. (2017). *Species Distribution Modeling With R.* Available at: https://cran.r-project.org/web/packages/dismo/vignettes/sdm.pdf (accessed January 8, 2017).

[B26] Husson F. Le S. Pages J. (2010). *Exploratory Multivariate Analysis by Example Using R.* Florida: CRC Press.

[B27] Hutchinson M. Xu T. Houlder D. Nix H. McMahon J. (2009). *ANUCLIM 6.0 User’s Guide. Fenner School of Environment and Society.* Canberra: Australian National University.

[B28] IPCC and Core Writing Team (2014). in *Climate Change 2014: Synthesis Report. Contribution of Working Groups I, II and III to the Fifth Assessment Report of the Intergovernmental Panel on Climate Change*, eds Pachauri R. K. Meyer L. A. (Geneva: IPCC), 151.

[B29] Janowiak M. K. Swanston C. W. Nagel L. M. Brandt L. A. Butler P. R. Handler S. D. (2014). A practical approach for translating climate change adaptation principles into forest management actions. *J. For.* 112 424–433. 10.5849/jof.13-094

[B30] Joyce L. A. Blate G. M. McNulty S. G. Millar C. I. Moser S. Neilson R. P. (2009). Managing for multiple resources under climate change: national forests. *Environ. Manage.* 44 1022–1032. 10.1007/s00267-009-9324-6 19588192

[B31] Köcher P. Gebauer T. Horna V. Leuschner C. (2009). Leaf water status and stem xylem flux in relation to soil drought in five temperate broad-leaved tree species with contrasting water use strategies. *Ann. For. Sci.* 66:101 10.1051/forest/2008076

[B32] Kolář T. Čermák P. Trnka M. Žid T. Rybníček M. (2017). Temporal changes in the climate sensitivity of Norway spruce and European beech along an elevation gradient in Central Europe. *Agric. For. Meteorol.* 239 24–33. 10.1016/j.agrformet.2017.02.028

[B33] Kriticos D. J. Jarošik V. Ota N. (2014). Extending the suite of bioclim variables: a proposed registry system and case study using principal components analysis. *Methods Ecol. Evol.* 5 956–960. 10.1111/2041-210X.12244

[B34] Kriticos D. J. Webber B. L. Leriche A. Ota N. Macadam I. Bathols J. (2012). CliMond: global high resolution historical and future scenario climate surfaces for bioclimatic modelling. *Methods Ecol. Evol.* 3 53–64.

[B35] Kržič A. Tošić I. Djurdjević V. Veljovic K. Rajković B. (2011). Changes in climate indices for Serbia according to the SRES-A1B and SRES-A2 scenarios. *Clim. Res.* 49 73–86. 10.3354/cr01008

[B36] Kuhn M. (2008). Building predictive models in R using the caret package. *J. Stat. Softw.* 28 1–26.27774042

[B37] Kunz J. Löffler G. Bauhus J. (2018). Minor European broadleaved tree species are more drought-tolerant than *Fagus sylvatica* but not more tolerant than *Quercus petraea*. *For. Ecol. Manag.* 414 15–27. 10.1016/j.foreco.2018.02.016

[B38] Lê S. Josse J. Husson F. (2008). FactoMineR: an R package for multivariate analysis. *J. Stat. Softw.* 25 1–18.

[B39] Lei J. Chen L. Li H. (2017). Using ensemble forecasting to examine how climate change promotes worldwide invasion of the golden apple snail (*Pomacea canaliculata*). *Environ. Monit. Assess.* 189:404. 10.1007/s10661-017-6124-y 28726175

[B40] Littell J. S. Peterson D. L. Millar C. I. Halloran K. A. O. (2011). U.S. national forests adapt to climate change through science–management partnerships. *Clim. Chang.* 110 269–296. 10.1007/s10584-011-0066-0

[B41] Loecher M. Ropkins K. (2015). RgoogleMaps and loa: unleashing R graphics power on map tiles. *J. Stat. Softw.* 63 1–18. 10.18637/jss.v063.i04

[B42] Mátyás C. Berki I. Czúcz B. Gálos B. Móricz N. Rasztovits E. (2010). Future of beech in southeast Europe from the perspective of evolutionary ecology. *Acta. Silvat. Lignar. Hungar.* 6 91–110.

[B43] Mausolf K. Wilm P. Hardtle W. Jansen K. Schuldt B. Sturm K. (2018). Higher drought sensitivity of radial growth of European beech in managed than in unmanaged forests. *Sci. Total Environ.* 642 1201–1208. 10.1016/j.scitotenv.2018.06.065 30045501

[B44] McIlroy D. (2018). *Packaged for R by Ray Brownrigg and Thomas P Minka, Transition to Plan 9 Codebase by Roger Bivand.* Available at: https://cran.r-project.org/web/packages/mapproj/mapproj.pdf

[B45] Peñuelas J. Ogaya R. Boada M. Jump S. A. (2007). Migration, invasion and decline: changes in recruitment and forest structure in a warming-linked shift of European beech forest in Catalonia (NE Spain). *Ecography* 30 829–837. 10.1111/j.2007.0906-7590.05247.x

[B46] Reif A. Xystrakis F. Gärtner S. Sayer U. (2017). Floristic change at the drought limit of European beech (*Fagus sylvatica L*). to Downy oak (*Quercus pubescens*) forest in the temperate climate of Central Europe. *Not. Bot. Horti. Agrobot. Cluj. Napoca.* 45 646–654. 10.15835/nbha45210971

[B47] Scharnweber T. Manthey M. Criegee C. Bauwe A. Schröder C. Wilmking M. (2011). Drought matters—declining precipitation influences growth of *Fagus sylvatica* L. and *Quercus robur* L. in north-eastern Germany. *For. Ecol. Manag.* 262 947–961. 10.1016/j.foreco.2011.05.026

[B48] Sedmáková D. Sedmák R. Bosela M. Ježík M. Blaženec M. Hlásny T. (2019). Growth–climate responses indicate shifts in the competitive ability of European beech and Norway spruce under recent climate warming in East–Central Europe. *Dendrochronologia* 54 37–48. 10.1016/j.dendro.2019.02.001

[B49] Serra-Diaz J. M. Ninyerola M. Lloret F. (2012). Coexistence of *Abies alba* (Mill).—*Fagus* sylvatica (L). and climate change impact in the Iberian Peninsula: a climatic-niche perspective approach. *Flora* 207 10–18. 10.1016/j.flora.2011.10.002

[B50] Seynave I. Gégout J.-C. Hervé J.-C. Dhôte J.-F. (2008). Is the spatial distribution of European beech (*Fagus sylvatica* L). limited by its potential height growth? *J. Biogeogr.* 35 1851–1862. 10.1111/j.1365-2699.2008.01930.x

[B51] Silva L. C. R. Sun G. Zhu-barker X. Liang Q. Wu N. Horwath W. R. (2016). Tree growth acceleration and expansion of alpine forests: the synergistic effect of atmospheric and edaphic change. *Sci. Adv.* 2:e1501302. 10.1126/sciadv.1501302 27652334 PMC5020709

[B52] Stein B. A. Glick P. Edelson N. Staudt A. (eds) (2014). *Climate-Smart Conservation: Putting Adaptation Principles Into Practice.* Washington, DC: National Wildlife Federation.

[B53] Takolander A. Hickler T. Meller L. Cabeza M. (2019). Comparing future shifts in tree species distributions across Europe projected by statistical and dynamic process-based models. *Reg. Environ. Change* 19 251–266. 10.1007/s10113-018-1403-x

[B54] Thuiller W. Lafourcade B. Engler R. Araújo M. B. (2009). BIOMOD—a platform for ensemble forecasting of species distributions. *Ecography* 32 369–373. 10.1111/j.1600-0587.2008.05742.x

[B55] Thuiller W. Münkemüller T. (2010). “Habitat suitability modeling,” in *effects of Climate Change on Birds*, eds Moller A. P. Fiedler W. Berthold P. (New York, NY: Oxford University Press), 77–85.

[B56] Thurm E. Hernández L. Baltensweiler A. Rasztovits E. Bielak K. Zlatanov T. (2018). Alternative tree species under climate warming in managed European forests. *For. Ecol. Manag.* 430 485–497. 10.1016/j.foreco.2018.08.028

[B57] Tian X. Sohngen B. Kim J. Ohrel S. Cole J. (2016). Global climate change impacts on forests and markets. *Environ. Res. Lett.* 11:035011 10.1088/1748-9326/11/3/035011

[B58] Urli M. Porté A. Cochard H. Guengant Y. Burlett R. Delzon S. (2013). Xylem embolism threshold for catastrophic hydraulic failure in angiosperm trees. *Tree Physiol.* 33 672–683. 10.1093/treephys/tpt030 23658197

[B59] Vose J. M. Peterson D. L. Patel-Weynand T. (2012). *Effects of Climatic Variability and Change on Forest Ecosystems: a Comprehensive Science Synthesis for the U.S. Gen. Tech. Rep. PNW-GTR-*870. Portland, OR: Department of Agriculture, Forest Service, Pacific Northwest Research Station.

[B60] Wickham H. (2016). *ggplot2: Elegant Graphics for Data Analysis.* New York, NY: Springer International Publishing.

[B61] Xu Z. Peng H. Peng S. (2015). The development and evaluation of species distribution models. *Acta. Ecol. Sin.* 35 557–567. 10.5846/stxb201304030600

[B62] Yao Y. Zhang B. (2015). The mass elevation effect of the tibetan plateau and its implications for alpine treelines. *Int. J. Climatol.* 35 1833–1846. 10.1002/joc.4123

[B63] Zang C. Hartl-meier C. Dittmar C. (2014). Patterns of drought tolerance in major European temperate forest trees: climatic drivers and levels of variability. *Glob. Change Biol.* 20 3767–3779. 10.1111/gcb.12637 24838398

[B64] Zimmermann J. Hauck M. Dulamsuren C. Leuschner C. (2015). Climate warming-related growth decline affects *Fagus sylvatica*, but not other broad-leaved tree species in Central European mixed forests. *Ecosystems* 18 560–572. 10.1007/s10021-015-9849-x

